# Impulsivity and pathological gambling: Is it a state or a trait problem?

**DOI:** 10.1186/1756-0500-4-492

**Published:** 2011-11-13

**Authors:** Florence DM Lai, Alison KY Ip, Tatia MC Lee

**Affiliations:** 1 Laboratory of Neuropsychology, The University of Hong Kong, Pokfulam Road, Hong Kong, China; 2 Laboratory of Cognitive Affective Neuroscience, The University of Hong Kong, Pokfulam Road, Hong Kong, China; 3 The State Key Laboratory of Brain and Cognitive Sciences, The University of Hong Kong, Pokfulam Road, Hong Kong, China

## Abstract

**Background:**

This study tested 37 Chinese male pathological gamblers and 40 controls to understand the relationship between pathological gambling and impulsivity as a long-term trait or a short-term state in the cognitive and affective domain.

**Results:**

Trait impulsivity was measured by the Barratt Impulsiveness Scale-11. State impulsivity in the cognitive and affective domains were measured by the Stroop Color Word Test and the Emotional Conflict Task, respectively. The pathological gamblers scored significantly higher than the controls on the Barratt Impulsiveness Scale-11. However, there were no significant group differences in performance on the Stroop Color Word Test or the Emotional Conflict Task.

**Conclusions:**

Findings clearly show that pathological gambling is associated with trait but not state impulsivity. In other words, pathological gambling is associated with an impulsivity stemming from enduring personality characteristics that lead gamblers to focus on short-term gains (trait impulsivity) rather than momentary cognitive or affective disinhibition (state impulsivity). Interventions should aim to change pathological gamblers' habitual functioning style by cultivating healthy reflection habits and focusing on long-term rewards.

## Background

Concern has been growing in recent years about the socio-economic impact pathological gambling has on society. According to the American Psychiatric Association [1], pathological gambling is characterized by persistent and recurrent maladaptive gambling behaviour, accompanied by disruptive consequences for familial, occupational, and social functioning. The lifetime prevalence rate is 1% to 2% in the United States and Canada [2]. In Hong Kong, 1.8% of residents can be classified as pathological gamblers according to DSM-IV-TR [3]. With the increasing recognition of pathological gambling as a psychiatric disorder, there has been an upsurge of academic studies investigating its pathophysiological mechanisms.

Since the 1980s, pathological gambling has been considered a disorder and categorized as an impulse control disorder in the DSM-IV-TR. The core features of pathological gambling are described as prolonged tolerance, withdrawal and craving, difficulty quitting, and major interference in life functioning. Rugle and Melamed [4] reported deficits in executive aspects of attention among pathological gamblers. Potenza [5] suggested that pathological gambling is a form of behavioural addiction, underlain by high impulsivity. In a recent review by Verdejo-Garcia and colleagues [6], impulsivity was addressed as an endophenotype of individuals at risk for substance use disorder and pathological gambling. The findings of these studies suggest a close relationship between impulsivity and pathological gambling.

Borrowing the state-trait anger theory [7], impulsivity could be viewed to have two sub-constructs: state impulsivity and trait impulsivity. State refers to transitory state at a particular time in response to a particular event, while trait refers to an enduring personality characteristic that describes or determines an individual's behaviour across a range of situations [8]. In other words, traits are long-term, while states are short-term. Swann and colleagues [9] employed a similar division of impulsivity in understanding antisocial personality disorder by using the Barratt Impulsiveness Scale Version 11 (BIS-11) [10] to measure the trait impulsivity of a group of antisocial personality disorder subjects. Their findings suggested dissociation between state and trait impulsivity. Christodoulou and colleagues [11] studied the relationship between trait impulsivity by the BIS-11 and state impulsivity by response disinhibition in the Hayling sentence completion task in a group of people with bipolar disorder. While the authors did not find any significant relationships between both tests, state and trait impulsivity appear to be orthogonal.

Glicksohn and Zilberman [12] conducted a study to examine whether gamblers will perform better than non-gamblers on the Iowa Gambling Task. Forty-two gamblers and 42 non-gamblers were recruited. Among the gamblers, 36% exhibited performance indicative of learning while 64% indicated a lack of learning. As for the non-gamblers group, 57% exhibited performance indicative of learning and 43% indicated a lack of learning. The authors concluded that the gamblers whose performance indicated a lack of learning in the task were more impulsive in trait. The authors assessed trait impulsivity by BIS-11 and trait-impulsivity was found to be predictive of performance on gambling task. This study hinted a possibility that pathological gamblers are more impulsive in trait than the healthy population.

State impulsivity can be attributed differently by attentional biases towards affectively neutral (cognitive domain) or affective events (affective domain) [13]. According to DSM-IV-TR [1], Impulse control disorders have a common major feature: "failure to resist an impulse, drive, or temptation to perform an act that is harmful to the person or to others" (p. 663). For that reason, we measured the state-impulsivity by Stroop Color Word Test (Stroop) [14] and Emotional Conflict Task (ECT) [15] as they capture the pathological gamblers' momentarily ability to inhibit interference, some undesired information with neutral [13] or affective valence [15]. Previous studies using the Stroop task have reported impaired performance in pathological gamblers [16,17]. Several research groups [18,19] have also reported that pathological gamblers perform worse on the Stroop task, relative to the controls. Though Potenza and co-workers [20] did not observe any significantly difference between pathological gamblers and controls in the task performance in Stroop, their functional imaging data revealed that during the Stroop task, pathological gamblers had hypoactivation in brain regions involved in decision-making and reward evaluation. For state impulsivity in the affective domain, the Emotional Conflict Task (ECT) [15] has been previously adopted to study emotional conflicts among healthy volunteers [21]. Task performance in ECT was found to be correlated strongly with the activation of brain regions involved in resolving emotional impulsivity. Furthermore, researchers using paradigms similar to the ECT observed that people with higher trait-anxiety had worse performance on the task [22]. The state-impulsivity in the affective domain has never been investigated among pathological gamblers. It is therefore worthwhile to explore into this area and see if it is one of the factors associated with pathological gambling.

Given the literature discussed above, it is reasonable to hypothesize that pathological gambling relates to trait and/or state impulsivity, such that people with pathological gambling would score higher than their healthy peers on measures of either or both types of impulsivity. The insight into the type(s) of impulsivity associated with pathological gamblers could inform the pathophysiological mechanisms initiating and maintaining the uncontrolled gambling behaviours. This study is the first to examine state impulsivity for pathological gamblers in the affective domain, measured here by the Emotional Conflict task.

## Methods

### Participants

This study was approved by the Institutional Review Board of the University of Hong Kong/Hospital Authority Hong Kong West Cluster. This study was conducted in accordance with the Principles of Helsinki. Pathological gamblers (*n *= 37) were recruited from a centre subsidized by government funding that treats pathological gamblers. The controls (*n *= 40) were recruited from the community by recruitment campaigns, including posters, emails and words of mouth, launched in the University of Hong Kong. Subjects, all of whom were male, were included if they were aged 25 to 50 (when the frontal lobe functions are of peak level in general [23]). The treatment rendered by the centre is mainly cognitive-behavioural therapy and all their clients are self-referred. The length of treatment should be around 1 year and most of the clients also sustain depression.

While depressive mood might affect individuals' impulsivity in the affective domain, e.g. performance in an emotional-stroop task [24], people with moderate to severe depression, those scored 29 or higher on the Beck Depression Inventory Version 2 (BDI-II), were excluded from participation. All participants were required to have normal or corrected-to-normal vision and to be free of neurological, psychiatric, or psychological disorders other than pathological gambling.

The South Oaks Gambling Screen (SOGS) [25] was used to screen for pathological gambling. The subjects in the pathological gamblers group met the criteria for pathological gambling and scored five or higher on the South Oaks Gambling Screen (SOGS) [25]. The Raven's Progressive Matrices Test was administered as a control of the general intelligence among participants, so that findings on the between-group comparison were not being confounded by attention deficits or comprehension ability associated with below-average general intelligence. The structured clinical interview from the DSM-IV was used for pathological gamblers groups for screening on co-morbidity, and the same interview for DSM-IV Axis I Disorders (SCID-I) was adopted for controls. Informed consents for participation were obtained from all participants of this study.

### Measures

All measurements and thier instructions provided are in Chinese.

#### Barratt Impulsiveness Scale Version 11 (BIS-11)

The BIS-11 is a 30-item self-report questionnaire with three subscales, namely, attentional impulsiveness, motor impulsiveness, and motor planning. Internal consistencies of .82 for the English version [10] and .80 for the Chinese version [26] were obtained for this scale, demonstrating this scale has good reliability.

#### Beck Depression Inventory (BDI-II)

The BDI-II is a self-reported inventory for initial assessment for depression. The inventory comprised of 21 multiple-choice questions. These questions focus on the general depression and somatic disturbances. The Chinese version was found to demonstrate high internal consistency (Cronbach α = .86) [27].

#### South Oaks Gambling Screen (SOGS)

The SOGS is a self-administered paper and pencil questionnaire with 20 items. The questionnaire was developed based on DSM-III criteria for pathological gambling [25]. Though it was found that SOGS may overestimate the number of pathological gamblers, the probability of identifying one as pathological gamblers was slim if the SOGS score was below 8 (the cut-off scores adopted in the current study). The Chinese version of SOGS was recorded with an acceptable internal consistency (Cronbach α = 0.69) [28].

#### Raven's Standard Progressive Matrix

Raven's Standard Progressive Matrix is a non-verbal multiple choice test with 60 items. The test aims to measure individual's general intelligence and non-verbal reasoning ability [29,30]

#### Stroop Color and Word Test (Stroop)

This study used a previously validated Chinese translation of the Victoria version of the Stroop Color and Word Test (Stroop) [14]. The Stroop test consists of three subtests: color dots (D), color of non-color words (W), and color words that conflict with the color in which they are presented (C). Printed stimuli were presented to the participants. Reaction times and accuracy were recorded separately for each subtest. The magnitude of the Stroop effect is defined as the difference between the reaction times in subtests C and D (interference score).

#### Emotional Conflict Task (ECT)

Adopted from the research of Etkin and colleagues [15], the emotional conflict task presents pictures of facial expressions with congruent or incongruent emotion words displayed across the face. Participants have to identify the facial expression, ignoring the written word. The task consisted of 148 stimuli divided into four blocks. A practice block of 25 trials was presented prior to the experimental blocks. A resting period was given between each block, wherein participants could rest as long as they hoped. The faces were either happy or fearful expressions drawn from Ekman and Friesen's set of emotion faces [31]. The faces were cropped and the words *fear *or *happiness *appeared in prominent red Chinese characters across each face, such that the word and expression were either congruent or incongruent.

Stimuli were presented in a pseudorandom order to ensure that there were approximately equal proportions of congruent-congruent, congruent-incongruent, incongruent-congruent, and incongruent-incongruent stimulus pairings. To avoid negative priming effects and repetition effects, no direction repetitions were presented of the same face with different words or exact face-word combinations [32]. Subjects were instructed to respond as accurately and quickly as possible by pushing response buttons corresponding to ''fear'' (right index finger) or ''happiness'' (right middle finger) based on the facial expressions. A sheet next to the keyboard clearly labeled the keys to minimize confusion. Stimuli were displayed using E-Prime (v. 1.0) on a 16-inch monitor. The output was the interference score, calculated as the reaction time in the incongruent condition minus the reaction time in the congruent condition.

### Procedure

The entire data-collection process lasted approximately two hours. The experimental tasks and screening measures were administered at the centre in a counterbalanced order.

## Results

### Demographics

One-way ANOVAs were performed to examine the group differences between the pathological gamblers and the control groups (Table 1). No significant between-group differences were reported on age, *F*(1, 75) = 0.246, *p *= 0.622; years of education, *F*(1, 75) = 2.07, *p *= 0.16; marital status, *χ*^2^(1) = 2.36, *p *= 0.31; employment, *χ*^2^(1) = 1.23, *p *= 0.27; nicotine use, *χ*^2^(1) = 0.01, *p *= 0.91; alcohol use, *χ*^2^(1) = 0.26, *p *= 0.61; and other substance use, *χ*^2^(1) = 0.90, *p *= 0.34; and Raven's scores, *F*(1, 75) = 3.1, *p *= 0.08. The pathological gamblers group scored higher than the control group on the SOGS, *F*(1, 75) = 833.19, *p *< 0.01. Despite our effort in screening out people with severe depression, pathological gamblers still scored significantly higher than controls in BDI-II, *F*(1, 75) = 22.07, *p *< 0.01. Given the focus of the current study is on pathological gambling rather than depression, we administered ANCOVA to partial out the effects of depression statistically.

**Table 1:** Demographic and clinical characteristics of pathological gamblers group and control group

	PG (*n *= 37)	Control (*n *= 40)		
	
	Mean (SD)	Mean (SD)	*F*(*df *= 1, 75)	*P *Value
Age	36.42 (6.90)	35.61 (7.31)	0.25	.622
Years of Education	11.66 (2.55)	13.60 (3.04)	0.21	.155
Raven's	48.81 (7.65)	51.7 (6.84)	3.06	.084
SOGS	14.30 (2.64)	1.05 (1.15)	833.19	<.001
BDI-II	14.46 (9.06)	6.55 (5.38)	22.07	<.001

			**χ^2 ^(*df *= 1)**	***P *Value**

Nicotine Use (%)	18.9	20	.01	.905
Alcohol Use (%)	10.8	7.5	.26	.614
Other Substance Use (%)	10.8	5	.90	.342

PG = pathological gambling group; Control = control group; BDI-II = Beck Depression Inventory Version 2; Raven's = Raven's Standard Progressive Matrices; SOGS = South Oaks Gambling Screen; "Other Substance Use" = the use of substances other than alcohol and nicotine.

A preliminary correlational analysis was performed with BDI-II total score and the following dependent variables: BIS-11 total score, Stroop interference score and ECT reaction time. A significant correlation was only found between BDI-II and BIS total score (*r *= .488, *p *<.001). Hence, one-way ANCOVAs were performed to examine the between-group differences with BDI-II total score as a covariate.

### Group Differences in Three Experimental Measures

#### Trait impulsivity

One-way ANCOVAs were performed to examine the group differences between the pathological gamblers and the control groups on the BIS-11 scale and total scores with BDI-II total score as covariate to partial out any possible effects due to depression (Table 2). The pathological gamblers group had significantly higher overall BIS-11 scores than the controls, *F*(1, 74) = 19.71, *p *< 0.01; BDI-II covariate: *F*(1, 74) = 5.74, *p *= 0.02. For the three BIS-11 subscales, since BDI-II score was not a significant covariate, student *t-*tests were performed to examine the possible differences between the pathological gamblers and the control group. Pathological gamblers scored significantly higher than the control group in all three BIS-11 subscales [Attentional Key: *t*(75) = 2.548, *P *= .013; Motor Key: *t*(75) = 3.206, *P *= .002; Non-Planning: *t*(75) = 2.684, *P *= .009].

**Table 2:** Task performance between pathological gamblers group and control group

	PG (*n *= 37)	Control (*n *= 40)
	
	Mean (SD)	Mean (SD)
Stroop		
Color-Dot (D) Accuracy (%)	99.89 (0.69)	100.00 (0.00)
Color-Word (C) Accuracy (%)	97.63 (3.87)	96.98 (5.50)
Color-Dot (D) Reaction Time (sec)	15.26 (9.58)	12.59 (4.37)
Color-Word (C) Reaction Time (sec)	27.13 (10.18)	23.80 (7.50)

ECT		
Congruent Accuracy (%)	92 (0.06)	90 (0.14)
Congruent Reaction Time (msec)	605.69 (67.66)	585.67 (84.40)
Incongruent Accuracy (%)	80 (0.15)	83 (0.16)
Incongruent Reaction Time (msec)	641.07 (67.13)	616.93 (83.98)

BIS-11 Total Score^a^	72.90 (9.50)	61.70 (6.23)
Attentional Key^b^	19.86 (3.61)	18.05 (2.36)
Motor Key^a^	22.95 (3.79)	20.25 (3.54)
Non-Planning Key^a^	29.27 (5.33)	26.13 (4.23)

PG = pathological gambling group; Control = control group. BIS-11 = Barratt Impulsiveness Scale Version 11; ECT = Emotional Conflict Task; Stroop = Stroop Color and Word Test; sec = seconds; msec = milliseconds.^a^Significant group difference found between pathological gambling group and control group by ANCOVA, *P *< 0.01.^b^Significant group difference found between pathological gambling group and control group by ANCOVA, *P *< 0.05.

#### State impulsivity - cognitive domain

The Stroop effect (i.e., interference) is calculated as the reaction time when reading the dot color (D) minus the reaction time when reading the color word (C). To analyze the interference, D minus C scores was entered into the model as a repeated measure in a two-way repeated measure ANOVA (Table 2). There were no significant group differences in terms of the overall interference, *F*(1, 75) = 3.13, *p *= 0.081. Participants were slower overall when reading the dot's color (D) than the color word (C). There was no significant interaction between group and interference, *F*(1, 75) = 0.19, *p *= 0.66. The non-significant interaction result implies that pathological gamblers do not have impaired attention ability.

#### State impulsivity - affective domain

There were no significant group differences in overall accuracy on the ECT, *F*(1, 75) = 0.02, *p *= 0.89, or in reaction time, *F*(1, 75) = 1.67, *p *= 0.20 (Table 2). Consistent with Etkin and colleague's study [29], reaction times on congruent trials were significantly faster than incongruent trials, *F*(1, 76) = 86.85, *p *< 0.01. A similar effect was also found in terms of accuracy, with significantly less correct responses on incongruent trials, *F*(1, 76) = 54.31, *p *< 0.01. The results suggest that the interference effect as measured by reaction time was not due to a speed-accuracy tradeoff.

Based on the disinhibition hypothesis of pathological gambling, it was expected that the pathological gamblers would show a greater interference effect. A two-way repeated measures ANOVA was conducted to analyze the effect of pathological gambling on reaction time. The interaction was non-significant, *F*(1, 75) = 1.36, *p *= 0.57. The non-significant interaction implies that pathological gambling does not affect participants' ability to inhibit automatic emotional responses.

## Discussion

This study examined the relationship between pathological gambling and impulsivity as both a trait and a state. The key finding was that the pathological gamblers differed from the healthy volunteers on the trait measure of impulsivity (BIS-11), but not on the state measures of impulsivity (the Stroop task & ECT). In other words, our findings provide partial support to the a priori hypothesis of this study and clearly suggest that trait and state impulsivity are independent within pathological gamblers.

Overall, the pathological gamblers were reported to have higher trait-impulsivity. Among the 30 items in BIS-11, the gamblers scored higher on items related to attitudes toward life, problem solving, and future planning, suggesting that these traits separate them from the control group. Pathological gamblers tended to be less future-oriented, less perseverant in problem solving, and more willing to endorse a happy-go-lucky attitude toward life over a more down-to-earth attitude. These impulsive traits might explain why they are more susceptible to pathological gambling.

Our findings on the trait-impulsivity are in line with findings on substance-abusers that drug addicts tend to make impulsive decisions and focus on short-term gains [33]. Drug addicts also have been found to have an impaired ability to inhibit negative interference from prior learning [34]. Indeed, drug dependence shares features with pathological gambling across the phenomenological, epidemiological, clinical, genetic, and biological domains [35-37]. Goudriaan and colleagues [38] reported that pathological gamblers and alcohol-dependent individuals had similar neurocognitive functioning. These findings support Potenza's suggestion that pathological gamblers and substance abusers share similar pathophysiological features and should fall under the umbrella of behavioural addiction [5].

In terms of state-impulsivity, our results show that state impulsivity in the cognitive domain, measured by the Stroop task, could not differentiate gamblers from the controls. Even though some studies found that pathological gamblers perform worse on the Stroop task [16,17], this inconsistency could be due to methodological differences between the studies. Though the above studies controlled for demographic and intelligence differences, some potentially important differences were not specified. For instance, the treatment that the gamblers were receiving and the respective stages of treatment may have an impact on the findings of these studies. This variability in treatment might cause discrepancies in the degree of momentary impulsivity. Also, the fact that our pathological gambling participants were receiving active treatment may account for the reduction in state impulsivity. This possibility could be verified in future studies of pathological gambling by recruiting gamblers before and after they begin treatment.

Similar to the findings of state impulsivity in the cognitive domain measured by the Stroop test, there were no significant group differences in state-impulsivity in the affective domain, measured by the ECT. The non-significant findings might imply that pathological gambling does not have any relationship with the gamblers' ability to inhibit automatic emotional responses. While impulsivity was suggested to be a common risk factor for both substance abuse and pathological gambling [6], Fishbein and colleagues' [39] found that substance abusers could control affective impulsivity as well as controls. Our findings further showed that pathological gamblers are similar to substance abusers that they are able to control their state-impulsivity in the affective domain when compared with controls.

Furthermore, as for the reaction time (RT) differences between pathological gamblers and controls on both Stroop task and ECT, it seems that pathological gamblers are overall slower than controls though such difference is insignificant. There is a trend that the pathological gamblers tend to use more time in inhibition while they are not especially impulsive in giving responses. It might then imply that pathological gamblers do not show a pronounce problem in state impulsivity. While cognitive behavioural treatment has seen the best empirical support for its efficacy in treating pathological gambling [40], it focuses mainly on treating the state impulsivity of pathological gamblers by teaching the gamblers how to resist the temptation to gamble at the moment the desire arises. Yet our findings indicate that pathological gamblers have impaired trait impulsivity--not state impulsivity. Therefore, cognitive behavioural interventions for pathological gamblers could be more focused on trait impulsivity, e.g. to cultivate long-term habits of reflection and more long-term reward assessments.

The present study has several limitations. In order to control gender confounds, only male subjects were recruited in this study, which limits the generalizability. Almost all pathological gambling studies face the challenge that the pathological gamblers in active treatment have abstained from pathological gambling during their treatment (i.e., three to six months). Our study is no exception. Hence, the current findings may not be generalized to gamblers actively engaged in gambling. Moreover, the absence of behavioural differences between pathological gamblers and controls on the Stroop test and ECT accuracy scores are possibly related to a ceiling effect. Future study may consider employing tasks of increased level of difficulty and provided data of different forms, e.g. neuro-imaging and physiological data as behavioural performance might not fully reflect the brain functions (see Potenza and coworkers [20]). Future studies may also look into the role of state-anxiety in relating to state- or trait-impulsivity for a more integrated view over different yet inter-related psychological mechanisms underlying the pathophysiology of pathological gambling.

## Conclusions

To conclude, impulsivity can be broken down into a state and a trait. Previous studies have closely linked impulsivity to pathological gambling, but the effects of trait and state impulsivity on pathological gamblers appear to be distinct. Indeed, pathological gambling seems to be related to an impulsive style of functioning and the cognitive and affective domains of state-impulsivity were not observed to be essential factors to differentiate pathological gamblers and controls.

## Abbreviations

DSM-IV-TR: the Diagnostic and Statistical Manual of Mental Disorders; BIS-11: Barratt Impulsiveness Scale Version 11; ECT: Emotional Conflict Task; BDI-II: Beck Depression Inventory Version 2; SOGS: South Oaks Gambling Screen; Stroop: Stroop Color and Word Test; W: non-color words; D: dot color; C: color word; RT: reaction time; SD: Standard Deviation; sec: seconds; msec: milliseconds.

## Competing interests

The authors declare that they have no competing interests.

## Authors' contributions

TMC conceptualized the research idea and designed the study. FDM administered the experiment and collected clinical data during clinical interviews. AKY performed statistical analysis. FDM wrote the first draft of the manuscript. TMC coordinated the research project and revised the manuscript critically. All authors contributed to and have approved the final manuscript.
